# Perceived psychological safety, state social connection, and student flourishing in collaborative music activities: a three-wave longitudinal study

**DOI:** 10.3389/fpsyg.2026.1956682

**Published:** 2026-09-25

**Authors:** Shihao Zhang

**Affiliations:** National Academy of Music “Prof. Pantcho Vladigerov”, Sofia, Bulgaria

**Keywords:** collaborative music activities, flourishing, perceived psychological safety, state social connection, three-wave longitudinal study, university music students

## Abstract

Collaborative music activities can support relationships and well-being, but the present study did not directly test music-specific mechanisms such as synchrony or collective expression. Instead, it examined a general interpersonal process within higher music education: whether students’ individually perceived psychological safety was longitudinally associated with flourishing through state social connection. A three-wave survey was completed by 341 university music students from 23 groups at three Chinese universities, with approximately 6 weeks between waves. Psychological safety was analyzed as an individual perception rather than as a shared team construct. Robust maximum likelihood estimation, full information maximum likelihood for partial missing data, cluster-robust standard errors, and 5,000 bootstrap samples were used. After baseline social connection and autoregressive effects were controlled, T1 perceived psychological safety was associated with higher T2 state social connection (*β* = 0.350, *p* < 0.001), which was associated with higher T3 flourishing after prior flourishing was controlled (*β* = 0.274, *p* < 0.001). The indirect effect was significant (B = 0.0949, *β* = 0.096, 95% BCa CI [0.054, 0.142]); the direct effect was not (*β* = −0.004, *p* = 0.941). Model comparisons did not uniquely support a one-way sequence: AIC favored a reciprocal model, BIC favored the more parsimonious hypothesized model, and prior social connection significantly predicted later perceived psychological safety. The findings therefore support a longitudinal indirect association alongside a plausible reciprocal process between perceived safety and connection. They should be understood as evidence for a general group-process model examined in a heterogeneous music-education context, not as evidence for a uniquely musical or causal mechanism.

## 1 Introduction

Collaborative music activities are among the most common forms of learning in higher music education. Choirs, ensembles, chamber groups, and group-based music courses require students to attend continuously to the sounds and actions of others while coordinating rhythm, timbre, musical interpretation, and stage expression. Music-making therefore becomes a highly interdependent social practice. The social bonding account of music proposes that shared rhythm, behavioral synchrony, and emotional coordination can strengthen interpersonal closeness and group cohesion (Savage et al., 2021). Previous studies have also shown that collective music-making can provide participants with a sense of belonging, social support, and opportunities for emotional expression (Barbeau et al., 2024; Levstek et al., 2021).

In positive psychology, flourishing denotes more than the absence of distress. It refers to functioning well across meaning and purpose, supportive relationships, competence, engagement, self-respect, and optimism (Keyes, 2002; Diener et al., 2010). In this study, flourishing is therefore treated as a broad indicator of positive psychological functioning rather than as momentary happiness.

The benefits of music participation do not emerge automatically in every setting. Students may experience shared creativity and mutual support during rehearsals, but they may also encounter public comparison, exposure of mistakes, peer evaluation, and unequal group roles. Professional music training often places strong demands on accuracy, discipline, and performance quality. These demands serve important educational purposes, yet they may also encourage some students to conceal difficulties, avoid asking for help, or remain silent when they hold different musical views. Recent research has argued that well-being in higher music education should not be understood solely in terms of individual coping. Course structures, institutional cultures, and interpersonal relationships also shape students’ experiences (Lubert et al., 2025). Among Chinese university music students, flourishing has likewise been associated with learning experiences and psychological resources (Yu and Xu, 2025).

Research on music and well-being has largely focused on participation frequency, performance anxiety, self-efficacy, or general social support. Less attention has been paid to the interpersonal conditions within collaborative activities. A recent review of music learning and well-being found that much of the existing evidence comes from qualitative studies and evaluations of specific programs, while the characteristics of activities, underlying mechanisms, and changes over time remain underexamined (Goopy and MacArthur, 2026). Although participation in group instrumental music has been linked to belonging, relationship development, and resilience, a more basic question remains unanswered: why do similar collaborative music activities help some students develop a sense of connection while leaving others feeling socially distant (de Bruin, 2025)?

### 1.1 Psychological safety in collaborative music activities

Psychological safety offers a useful framework for understanding these differences. Kahn (1990) originally described psychological safety as a condition in which people can express and engage themselves without fear of negative consequences, and Edmondson (1999) subsequently defined team psychological safety in terms of interpersonal risk-taking. Meta-analytic evidence further shows that psychological safety is associated with information sharing, learning, and performance across individual and group levels (Frazier et al., 2017). In the present study, the construct refers to each participant’s perception that questions, mistakes, disagreement, and requests for help can be expressed within a focal collaborative music activity without humiliation, exclusion, or punishment. It is analyzed as an individual perception rather than as an aggregated or shared group property.

Collaborative music activities involve many situations in which students must take interpersonal risks. They may need to admit that they have not mastered a passage, identify coordination problems during rehearsal, question an interpretation proposed by a teacher or conductor, or experiment with an unfamiliar artistic approach. When psychological safety is low, students may communicate less and adopt self-protective strategies. The group may still complete its rehearsal tasks, but genuine collaboration may remain limited. Educational research has linked psychological safety with students’ willingness to express ideas, share knowledge, manage uncertainty, and engage in creative activity (Han et al., 2022; Moffett et al., 2024). Research on interdisciplinary student teams has also shown that teaching arrangements and instructor behavior influence whether students are willing to speak up, raise concerns, and participate in collaborative problem-solving (Robinson and Held, 2025).

These processes are relevant to collaborative music learning because ensemble quality depends on members’ ability to identify problems, exchange feedback, and adjust their own behavior in response to others. Creative interpretation can also require tolerance of uncertainty and temporary imperfection. However, the present study did not directly measure synchrony, shared emotional experience, or collective expression. It therefore does not test whether a music-specific mechanism produced the observed associations. Rather, it examines a general interpersonal process in a setting where public error exposure, reciprocal adjustment, and artistic disagreement are common.

### 1.2 The mediating role of state social connection

Psychological safety may not directly alter students’ overall functioning. Its influence may first appear in the social experiences that develop within a particular group. State social connection refers to feelings of closeness, acceptance, and belonging experienced at a specific time and in a specific context. It differs from relatively stable social tendencies and from general evaluations of support from family, friends, or wider social networks. Research on the UBC State Social Connection Scale indicates that social connection varies across interactions and situations, making it suitable for assessing immediate relational experiences within a particular activity (Lok and Dunn, 2023).

This contextual sensitivity is especially important in music groups. A student may have supportive family relationships and close friendships while still feeling like an outsider in a particular ensemble. Another student may receive encouragement, assistance, and recognition during recent rehearsals and consequently experience a stronger sense of belonging. Participation frequency alone cannot explain these differences. State social connection provides a more direct indication of how students experience their current music group.

Psychological safety may provide the interpersonal foundation for social connection. When students can acknowledge mistakes, ask for help, or express different musical interpretations, the responses of other group members communicate acceptance, respect, and recognition of group membership. Repeated interactions of this kind can gradually strengthen closeness and belonging. Students who expect criticism or exclusion may instead restrict what they say, avoid seeking support, and focus on preventing negative evaluation. They may participate in the same physical space while remaining socially distant. Research on collective music-making suggests that supportive guidance, inclusive participation, and shared expression can help sustain relationships and group belonging (Levstek et al., 2021). Psychological safety may therefore strengthen state social connection by encouraging genuine communication and reciprocal interaction.

### 1.3 Social connection and flourishing

Flourishing refers to positive functioning across areas such as meaning in life, supportive relationships, competence, engagement, and optimism. It involves more than the absence of anxiety, depression, or stress. It also reflects whether individuals experience their lives as meaningful and are able to participate effectively in everyday activities. Social connection is closely related to flourishing because positive relationships provide support, affirm personal value, and create shared sources of meaning. Recent evidence suggests that interpersonal connection and prosocial orientation explain meaningful variation in flourishing (Rahe and Jansen, 2023).

A similar relationship can be observed in university learning environments. Large-scale research with university students has linked social development, value development, instructional support, and feedback with hedonic, eudaimonic, and social well-being (Zhao and Hua, 2025). Music learning also involves a strongly shared form of production. Rehearsals and performances depend on each member’s contribution as well as coordination among members. When students feel accepted, believe that their contribution matters, and work with others toward a shared musical outcome, the activity may acquire personal meaning beyond technical training.

The present study therefore evaluates a temporally ordered indirect pathway. Individually perceived psychological safety represents a person’s appraisal of the interpersonal conditions in a collaborative music activity. State social connection represents a proximal relational experience, and flourishing represents more distal positive functioning. Musical synchrony, shared goals, and collective expression may create opportunities for connection, but because these features were not measured directly, they serve only as contextual rationale rather than tested explanatory mechanisms.

### 1.4 Research gaps and the present study

Existing research has established important links among music participation, social relationships, and well-being, but several gaps remain. Much of the literature is cross-sectional, making it difficult to evaluate temporal ordering or possible reciprocity. Students who already feel connected may also perceive their groups as safer. Research on music and well-being often emphasizes participation amount and gives less attention to individual appraisals of interpersonal interaction. Few studies have examined longitudinal associations among perceived safety, context-specific connection, and broader positive functioning while controlling for construct stability. Recent reviews have therefore called for more precise investigation of the characteristics, mechanisms, and outcomes of different forms of music learning rather than treating music participation as a uniform intervention (Goopy and MacArthur, 2026).

The present study used a three-wave longitudinal design in which perceived psychological safety, state social connection, and flourishing were measured at intervals of approximately 6 weeks. Repeated measurement allowed prior levels of each construct to be controlled and permitted comparison of hypothesized, reverse, alternative-ordering, and reciprocal models. A random-intercept cross-lagged panel model was also used as an exploratory robustness analysis. The aim was not to establish causal priority, but to evaluate whether the proposed indirect sequence was compatible with the data and whether reciprocal relations remained plausible.

The study’s contribution is deliberately contextual rather than music-specific. It tests whether a general individual-level group-process model - perceived interpersonal safety, subsequent social connection, and later flourishing - is observable in higher music education, where feedback, public mistakes, coordination, and interpretation are recurrent features. The pooled sample included choirs, orchestras, Chinese instrumental ensembles, chamber groups, bands, group classes, pedagogy groups, and improvisation or composition groups. These settings may differ in leadership, size, hierarchy, repertoire, and interdependence; the present study was not designed or powered to estimate separate pathways for each type.

The overall objective was to determine whether perceived psychological safety was longitudinally associated with students’ flourishing through state social connection in collaborative music activities, while accounting for prior levels of the focal constructs and evaluating plausible alternative temporal orderings.

RQ1: To what extent does the proposed perceived-safety → state-connection → flourishing sequence account for longitudinal associations after autoregressive and baseline controls?

RQ2: Do reverse, alternative-ordering, or reciprocal models provide an equally or more plausible representation of the longitudinal relations?

Based on this framework, the following hypotheses were proposed:

*H1*: After controlling for T1 state social connection and relevant baseline variables, T1 perceived psychological safety will be positively associated with T2 state social connection.

*H2*: After controlling for prior flourishing, T2 state social connection will be positively associated with T3 flourishing.

*H3*: T2 state social connection will carry a significant longitudinal indirect association between T1 perceived psychological safety and T3 flourishing.

## 2 Materials and methods

### 2.1 Study design and participants

This study used a three-wave longitudinal survey design to examine associations among individually perceived psychological safety, state social connection, and flourishing among university music students engaged in collaborative music activities. Participants were recruited from 23 music-group clusters at three universities in China. Their focal activities were choir or choral courses (*n* = 78), orchestras (*n* = 53), Chinese instrumental ensembles (*n* = 32), chamber groups (*n* = 31), bands (*n* = 39), group vocal classes (*n* = 15), group instrumental classes (*n* = 26), music-pedagogy groups (*n* = 13), and collective improvisation or composition groups (*n* = 54). Eligibility required participants to be at least 18 years old, enrolled in a university music-related program, involved in at least one collaborative music activity at T1, and willing to complete follow-up surveys.

At T1, 378 questionnaires were collected. Following the exclusion of 37 responses based on prespecified data-quality criteria, the final analytic cohort comprised 341 participants, corresponding to a valid response rate of 90.2%. All 341 completed the three survey waves and the flourishing measure at every wave. Of these, 306 had all nine core scores, whereas 35 had partial missingness in one or more follow-up group-context measures. Participants with complete core data did not differ significantly from those with partial group-context data in T1 perceived psychological safety, state social connection, flourishing, age, gender, or years of music training.

All three surveys were administered through Wenjuanxing. Every member of the analytic cohort completed a survey at T1, T2, and T3. Consecutive waves were separated by approximately 6 weeks (T1-T2: M = 42.0 days; T2-T3: M = 42.2 days; range at each interval = 28–56 days). This interval was selected to cover several cycles of rehearsal, feedback, and interaction, allowing a proximal relational experience to change while reducing the likelihood that the focal activity context would be entirely different. It should be understood as a design window rather than evidence that 6 weeks is a universal causal lag. At each follow-up, a branching item asked whether the participant remained involved in the focal activity. Students reporting current participation or reduced-frequency participation completed the group-context measures. Those reporting a temporary stop, complete withdrawal, or a change to another group skipped perceived psychological safety, state social connection, and collaborative-activity items for that wave. Flourishing was completed by all 341 participants because it referred to general life functioning. Eligibility for the group-context block was reassessed at each wave, so missingness could be nonmonotonic if participation later resumed.

### 2.2 Measures

#### 2.2.1 Psychological safety

Perceived psychological safety was assessed using Edmondson's (1999) seven-item Team Psychological Safety Scale, with wording adapted to the focal collaborative music activity. The items asked whether the respondent personally felt able to acknowledge mistakes, raise concerns, seek help, and express different views. Responses used a seven-point Likert scale; PS1, PS3, and PS5 were reverse-scored. Scale scores were individual item means. No aggregation to the music-group level was performed. Cronbach’s alpha was 0.935, 0.946, and 0.947 at T1, T2, and T3, respectively.

#### 2.2.2 State social connection

State social connection was measured using the UBC State Social Connection Scale (Lok and Dunn, 2023). The scale underwent forward translation, back-translation, expert review, and adaptation to the collaborative music context. It consisted of 10 items rated on a seven-point Likert scale. Items SC1, SC2, SC3, and SC5 were reverse-scored. Cronbach’s alpha values across the three waves were 0.958, 0.960, and 0.959, respectively.

#### 2.2.3 Flourishing

Flourishing was assessed using the eight-item Flourishing Scale developed by Diener et al. (2010). The scale measures positive psychological functioning in areas such as meaning in life, positive relationships, competence, engagement, and optimism. All items were positively worded and rated on a seven-point Likert scale. Cronbach’s alpha values were 0.958 at T1, 0.954 at T2, and 0.951 at T3.

#### 2.2.4 Collaborative music activity characteristics and continued participation intention

Collaborative music activity characteristics (CM) were assessed using five self-developed frequency items covering shared discussion of musical expression, experimentation with new ideas, reciprocal feedback, and participation in group decision-making. CM was conceptualized as a potential baseline contextual antecedent or covariate, not as a mediator or moderator in the confirmatory model. Because it was measured concurrently with perceived psychological safety and social connection, causal ordering among these constructs could not be established. The scale was therefore used to describe the context and in a prespecified sensitivity analysis using T1 CM only.

Continued participation intention was measured at T3 for an exploratory analysis only. Its measurement and results have been moved to Supplementary Note S5 and are not used to support the confirmatory longitudinal hypotheses.

### 2.3 Data analysis

Data analyses were conducted in R using lavaan (Rosseel, 2012) and psych. Preliminary analyses included data screening, descriptive statistics, Cronbach’s alpha, McDonald’s omega, and pairwise-complete Spearman correlations with Holm-Bonferroni correction. The wide-format file was audited by unique participant ID and wave prefix. Reverse-scored items and all scale means were recomputed from the item-level data and compared with the stored scores before the correlation matrix was regenerated. Confirmatory factor analysis and tests of configural, metric, scalar, and strict longitudinal measurement invariance were then conducted for the three core measures.

The unit of analysis was the student. Psychological safety was interpreted as individually perceived psychological safety, not as a shared group-level construct. ICC(1) values were calculated only to describe clustering within the 23 music-group clusters. Cluster-robust standard errors were used as a sensitivity correction for dependence; they were not treated as evidence supporting aggregation.

The primary observed-score model included separate autoregressive paths for perceived psychological safety, state social connection, and flourishing across adjacent waves; covariances among the three T1 variables; residual covariances among the three variables within T2 and within T3; the focal paths T1 perceived psychological safety to T2 state social connection and T2 state social connection to T3 flourishing; a direct path from T1 perceived psychological safety to T3 flourishing; and baseline adjustment paths from T1 flourishing to T2 state social connection and from T1 flourishing and T1 state social connection to T3 flourishing. No equality constraints were imposed on the autoregressive paths in the primary model. Robust maximum likelihood estimation was applied, partial missing data were handled using FIML, and the indirect effect was evaluated using 5,000 bias-corrected and accelerated bootstrap samples in the ML re-estimation.

All competing models used the same nine observed variables, autoregressive backbone, wave-specific covariance structure, baseline adjustment logic, FIML treatment of missing data, and analytic sample. The reverse model specified T1 perceived psychological safety to T2 flourishing to T3 state social connection, with the corresponding direct path from T1 perceived psychological safety to T3 state social connection. The alternative-ordering model specified T1 state social connection to T2 perceived psychological safety to T3 flourishing, with the corresponding direct path from T1 state social connection to T3 flourishing. The reduced reciprocal model retained the hypothesized paths and added T1 state social connection to T2 perceived psychological safety and T2 flourishing to T3 state social connection. These two added paths account for its two fewer degrees of freedom. Information criteria were compared under directly comparable ML/FIML specifications (Burnham and Anderson, 2002).

Missing-data sensitivity was examined by comparing participants with and without wave-specific group-context measures on prior observed scores, by a complete-case regression-based mediation analysis among the 306 participants with all nine core scores, and by inverse-probability weighting for T2 group-context availability using baseline psychological safety, social connection, flourishing, age, music-training years, gender, and university. These diagnostics address associations with observed variables but cannot rule out missing-not-at-random mechanisms based on unobserved experiences. Additional robustness analyses used a RI-CLPM, university and timing adjustment, and cluster-robust standard errors.

### 2.4 Ethics statement

The study protocol was reviewed by the Scientific Research Ethics Committee of the National Academy of Music “Prof. Pantcho Vladigerov,” which determined on 10 May 2026 that the study met the criteria for exemption from full ethics review because it involved an anonymous, minimal-risk questionnaire survey of adult university students. Before completing the T1 survey, all participants received information about the purpose and procedures of the study, the voluntary nature of participation, confidentiality arrangements, and their right to withdraw at any stage without penalty, and provided electronically documented written informed consent. Responses across the three waves were linked using anonymous identification codes. All data were stored confidentially and used solely for academic research. The study was conducted in accordance with applicable institutional requirements and the principles of the Declaration of Helsinki.

## 3 Results

### 3.1 Descriptive statistics and reliability

Table 1 presents the descriptive statistics. All 341 participants completed each survey wave and all flourishing items. The group-context block was available for 341 participants at T1, 324 at T2, and 322 at T3. Across the nine core scores, 306 participants (89.7%) had complete data, while 35 had at least one missing group-context score. FIML incorporated all available information from the full analytic cohort. Figure 1 and Table 2 show that the missingness was wave-specific rather than conventional monotonic study attrition.

**Table 1:** Descriptive statistics of key variables.

Variable	*N*	M	SD	Min	Max	Skewness	Kurtosis
T1_PS_mean	341	3.984	1.126	1.0	7.0	0.053	−0.399
T2_PS_mean	324	4.004	1.147	1.0	7.0	0.008	−0.147
T3_PS_mean	322	4.026	1.143	1.286	6.857	−0.038	−0.471
T1_SC_mean	341	3.998	1.119	1.1	7.0	0.124	−0.159
T2_SC_mean	324	4.019	1.159	1.0	6.8	−0.064	−0.513
T3_SC_mean	322	3.975	1.141	1.1	7.0	0.092	−0.266
T1_FL_mean	341	3.946	1.163	1.125	6.75	−0.066	−0.317
T2_FL_mean	341	3.984	1.15	1.375	7.0	0.192	−0.378
T3_FL_mean	341	3.975	1.131	1.125	6.75	−0.052	−0.474
T1_CM_mean	341	3.789	0.975	1.0	5.0	−0.632	−0.493
T2_CM_mean	324	3.816	0.946	1.0	5.0	−0.743	0.136
T3_CM_mean	322	3.84	0.934	1.2	5.0	−0.569	−0.493

Variable-specific N reflects wave-specific measure availability (T1 *N* = 341; T2 perceived psychological safety/state social connection/collaborative music characteristics *N* = 324; T3 corresponding group-context measures *N* = 322; flourishing *N* = 341 at every wave). Complete cases across the nine core perceived-safety, connection, and flourishing scores: *N* = 306. PS, perceived psychological safety; SC, state social connection; FL, flourishing; CM, collaborative music activity characteristics. PS, SC, and FL were scored from 1 to 7; CM was scored from 1 to 5.

**Figure 1:**
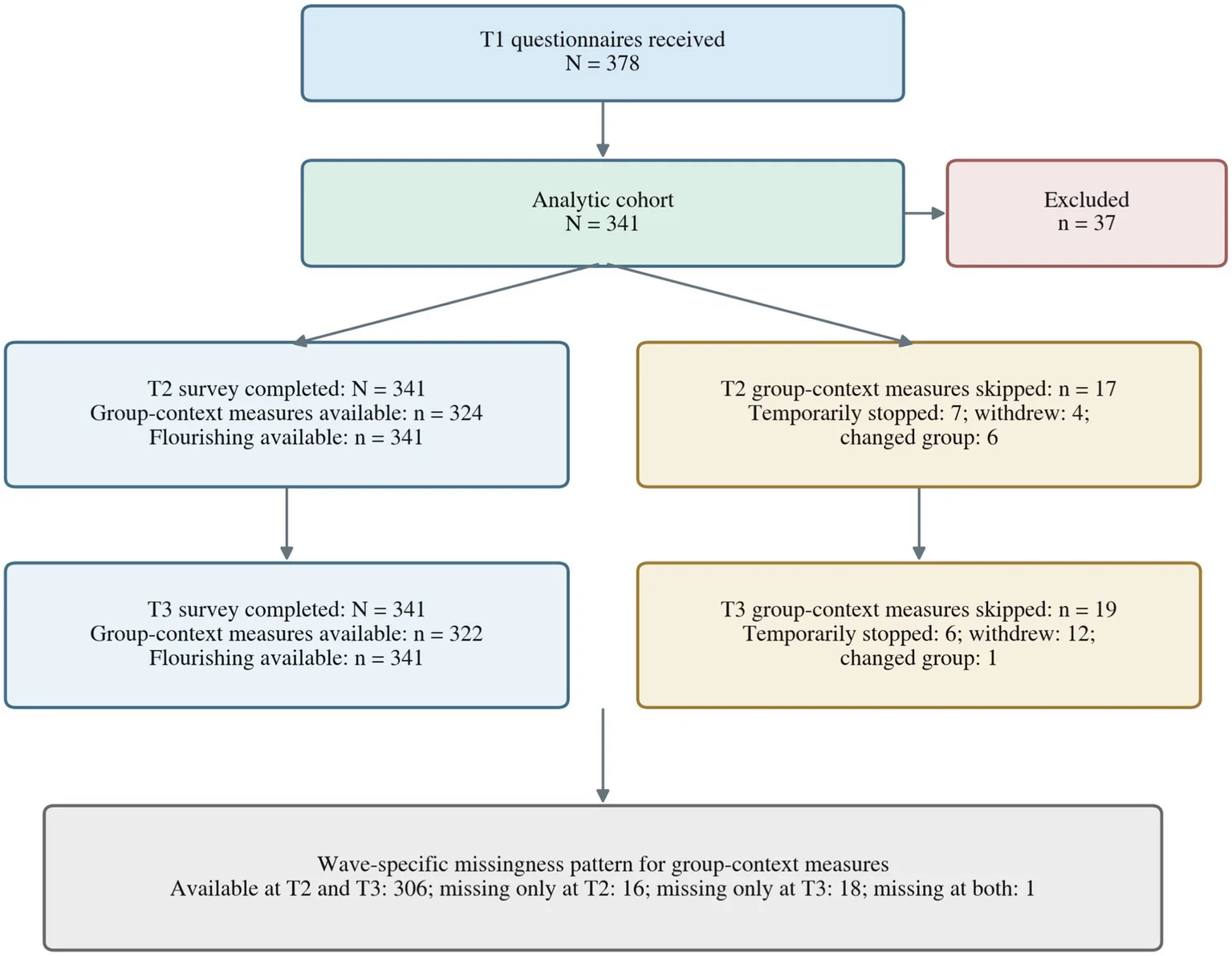
Participant flow and availability of group-context measures. All 341 participants completed the three survey waves and the flourishing measure; group-context measures were skipped according to wave-specific participation status.

**Table 2:** Wave-specific missingness pattern for group-context measures.

T2 group-context measures	T3 group-context measures	*n*	% of analytic cohort
Available	Available	306	89.7
Missing	Available	16	4.7
Available	Missing	18	5.3
Missing	Missing	1	0.3
Total		341	100.0

Group-context measures comprise perceived psychological safety, state social connection, and collaborative music activity characteristics. All participants completed flourishing at T2 and T3.

Scale means ranged from 3.79 for T1 CM to 4.03, and standard deviations ranged from 0.93 to 1.16, indicating sufficient individual variation in the sample. The absolute values of skewness were below 1, with a maximum of 0.74, and the absolute values of kurtosis were also below 1, with a maximum of 0.51, suggesting that the distributions were approximately normal. Cronbach’s alpha values for all scales exceeded 0.85 and ranged from 0.935 to 0.960, as shown in Table 3, indicating high internal consistency across the three waves. The generally high alpha values, most of which exceeded 0.91, may indicate some item redundancy or relatively narrow construct coverage. The structural validity of the measures was therefore examined further through confirmatory factor analysis.

**Table 3:** Scale reliability: Cronbach’s alpha and McDonald’s omega across three waves.

Scale	Wave	Items	Cronbach_Alpha	Evaluation	Omega_N	McDonald_Omega_Total
Psychological Safety	T1	7	0.935	excellent	341	0.935
Psychological Safety	T2	7	0.946	excellent	324	0.947
Psychological Safety	T3	7	0.947	excellent	322	0.947
State Social Connection	T1	10	0.958	excellent	341	0.958
State Social Connection	T2	10	0.96	excellent	324	0.959
State Social Connection	T3	10	0.959	excellent	322	0.959
Flourishing	T1	8	0.958	excellent	341	0.958
Flourishing	T2	8	0.954	excellent	341	0.954
Flourishing	T3	8	0.951	excellent	341	0.952
Collaborative Music	T1	5	0.914	excellent	341	0.915
Collaborative Music	T2	5	0.918	excellent	324	0.918
Collaborative Music	T3	5	0.912	excellent	322	0.913

All Cronbach’s alpha values were at least 0.85. Omega values were computed using the psych package in R.

#### 3.1.1 Follow-up participation and missingness

The 35 participants with partial missing data at T2 or T3 did not differ significantly from the 306 participants with complete data on the T1 core variables. For T1 psychological safety, the mean was 3.98 (SD = 1.14) in the complete-data group and 4.00 (SD = 1.02) in the partial-missing-data group, *t* = −0.06, *p* = 0.950, d = −0.01. For T1 state social connection, the corresponding means were 3.99 (SD = 1.13) and 4.06 (SD = 0.99), *t* = −0.35, *p* = 0.728. For T1 flourishing, the means were 3.94 (SD = 1.16) and 4.00 (SD = 1.16), *t* = −0.29, *p* = 0.771. The two groups also did not differ significantly in age (*p* = 0.914), gender (*p* = 0.717), or years of music training (*p* = 0.508).

However, the proportion of missing data differed significantly across universities, χ^2^(2) = 13.60, *p* = 0.001. The missing-data rates were 7.5% at University 1, 5.7% at University 2, and 20.5% at University 3. To reduce the possibility of university-level confounding, university dummy variables were included in the sensitivity analysis described in Section 3.9.

The availability pattern was 306 participants with group-context scores at both T2 and T3, 16 missing only at T2, 18 missing only at T3, and one missing at both waves (Table 2). At T2, the 17 skipped group-context blocks comprised seven temporary stops, four complete withdrawals, and six group changes. At T3, the 19 skipped blocks comprised six temporary stops, 12 complete withdrawals, and one group change. Of the 17 participants who skipped the T2 block, 13 reported active participation and three reduced-frequency participation at T3; this accounts for the nonmonotonic pattern. Flourishing remained available for all 341 participants because it was administered outside the group-context branch.

#### 3.1.2 Clustering and level of analysis

ICC(1) values for perceived psychological safety were 0.016 at T1, 0.014 at T2, and 0.005 at T3; corresponding values for state social connection were 0.023, 0.008, and 0.030. These small coefficients indicate limited between-cluster variance and do not support treating either score as an aggregated shared belief in this dataset. The individual-level interpretation was therefore retained, with cluster-robust standard errors used only to account for residual dependence.

### 3.2 Correlation analysis

The Spearman correlation matrix is presented in Supplementary Table S1, and exact pairwise sample sizes are reported in Supplementary Table S2. Pairwise-complete observations were used. Depending on the variables involved, *N* ranged from 306 to 341: correlations involving a T2 group-context score used *N* = 324 unless paired with a T3 group-context score (*N* = 306), and correlations involving a T3 group-context score used *N* = 322 unless paired with a T2 group-context score. Repeated measures of the same construct showed moderate to strong temporal stability: perceived psychological safety, *r* = 0.539–0.545; state social connection, *r* = 0.647–0.649; and flourishing, *r* = 0.500–0.581.

The two focal lagged correlations were T1 perceived psychological safety with T2 state social connection, *r* = 0.577, *N* = 324, adjusted *p* < 0.001, and T2 state social connection with T3 flourishing, *r* = 0.420, *N* = 324, adjusted *p* < 0.001. Both exceeded the corresponding contemporaneous correlations (*r* = 0.426 at T1 and *r* = 0.235 at T2, respectively). Because this pattern is unusual, wave labels, unique-ID alignment, reverse scoring, and scale-mean calculations were rechecked against the item-level file. Every stored scale mean matched the independently recomputed mean, and the same correlations were reproduced. The coefficients were therefore retained, but they are treated as descriptive bivariate associations rather than evidence of temporal direction.

### 3.3 Confirmatory factor analysis

Table 4 presents the CFA fit indices for each wave. At all three time points, the three-factor model, in which psychological safety, state social connection, and flourishing were specified as separate factors, provided a better fit than the single-factor Harman model. The three-factor model showed borderline fit at T1 (CFI = 0.947, RMSEA = 0.067) and good fit at T2 (CFI = 0.976, RMSEA = 0.044) and T3 (CFI = 0.982, RMSEA = 0.038).

**Table 4:** Confirmatory factor analysis fit indices (three waves).

Wave	Model	Chi^2^	df	*p*	CFI	TLI	RMSEA	RMSEA_CI	SRMR	AIC	BIC	Converged
T1	3-Factor	693.71	272	0	0.947	0.941	0.067	[0.061, 0.074]	0.043	21502.6	21801.5	True
T1	1-Factor (Harman)	4322.55	275	0	0.491	0.444	0.208	[0.202, 0.213]	0.2	25125.4	25412.8	True
T2	3-Factor	453.05	272	0	0.976	0.974	0.044	[0.037, 0.051]	0.042	20794.4	21093.3	True
T2	1-Factor (Harman)	4455.16	275	0	0.45	0.4	0.211	[0.206, 0.217]	0.236	24790.5	25077.9	True
T3	3-Factor	403.27	272	0	0.982	0.98	0.038	[0.030, 0.045]	0.034	20660.4	20959.3	True
T3	1-Factor (Harman)	4528.38	275	0	0.425	0.372	0.213	[0.208, 0.218]	0.253	24779.5	25066.9	True

MLR estimator. 3-Factor model separates PS, SC, and FL. 1-Factor Harman model tests common method variance. CFI, Comparative Fit Index; TLI, Tucker-Lewis Index; RMSEA, Root Mean Square Error of Approximation; SRMR, Standardized Root Mean Square Residual.

By contrast, the single-factor model showed poor fit at all three waves (T1: CFI = 0.491; T2: CFI = 0.450; T3: CFI = 0.425) and performed substantially worse than the three-factor model. These results suggest that a single common factor was unlikely to account for all observed covariance. However, because all core variables were assessed through self-report measures, the possible influence of common method bias cannot be ruled out entirely. The temporal separation of the three waves may have reduced this risk to some extent (Podsakoff et al., 2003), but it remains a limitation of the study.

Standardized factor loadings ranged from 0.733 to 0.907 and were all above 0.70. Composite reliability values ranged from 0.935 to 0.959.

Supplementary Table S3 presents the average variance extracted (AVE) and composite reliability (CR) values for each factor across the three waves. CR values exceeded 0.90 for all factors (PS: 0.935–0.947; SC: 0.958–0.959; FL: 0.952–0.958), well above the recommended threshold of 0.70 (Bagozzi and Yi, 1988), indicating strong composite reliability. AVE values ranged from 0.673 to 0.721 for PS, from 0.694 to 0.704 for SC, and from 0.712 to 0.741 for FL. All values exceeded the recommended criterion of 0.50 (Fornell and Larcker, 1981).

The square roots of AVE for each factor (PS: 0.821–0.849; SC: 0.833–0.839; FL: 0.844–0.861) were greater than the correlations between that factor and the other constructs, supporting discriminant validity. Although the high alpha values (> 0.91) indicated strong internal consistency, the combination of AVE values approaching 0.70 and high CR values may suggest some limitations in the breadth of content covered by the scales. Overall, the CFA, AVE, and CR results consistently supported the convergent and discriminant validity of the measures.

### 3.4 Longitudinal measurement invariance

As shown in Table 5, all three measures—psychological safety, state social connection, and flourishing—achieved scalar invariance, while some also supported strict invariance. Based on the criteria recommended by Chen (2007), namely ΔCFI ≥ − 0.010 and ΔRMSEA ≤ 0.015, the changes in CFI and RMSEA remained within acceptable limits across the nested models. Establishing scalar invariance indicates that the measures assessed the same constructs across the three waves and that their item intercepts were equivalent over time. This provided a measurement basis for subsequent longitudinal comparisons using observed scale scores.

**Table 5:** Longitudinal measurement invariance across three waves.

Scale	Model	Chi^2^	df	CFI	TLI	RMSEA	RMSEA_CI	SRMR	Delta_CFI	Delta_RMSEA	Delta_SRMR	Decision
Psychological Safety	Configural	293.74	168	0.979	0.974	0.047	[0.038, 0.056]	0.079				Baseline
Psychological Safety	Metric	288.36	177	0.981	0.978	0.043	[0.034, 0.052]	0.035	0.002	−0.004	−0.044	PASS
Psychological Safety	Scalar	297.63	189	0.982	0.98	0.041	[0.032, 0.050]	0.035	0.001	−0.002	0.0	PASS
Psychological Safety	Strict	330.51	203	0.979	0.978	0.043	[0.034, 0.051]	0.036	−0.003	0.002	0.001	PASS
State Social Connection	Configural	973.58	375	0.941	0.931	0.068	[0.063, 0.074]	0.049				Baseline
State Social Connection	Metric	991.45	390	0.94	0.934	0.067	[0.062, 0.072]	0.052	−0.001	−0.001	0.003	PASS
State Social Connection	Scalar	1005.53	408	0.941	0.937	0.066	[0.060, 0.071]	0.052	0.001	−0.001	0.0	PASS
State Social Connection	Strict	1018.55	428	0.942	0.941	0.064	[0.059, 0.069]	0.053	0.001	−0.002	0.001	PASS
Flourishing	Configural	254.53	228	0.997	0.996	0.018	[0.000, 0.030]	0.116				Baseline
Flourishing	Metric	242.78	239	1.0	0.999	0.007	[0.000, 0.023]	0.028	0.003	−0.011	−0.088	PASS
Flourishing	Scalar	264.79	253	0.998	0.998	0.012	[0.000, 0.025]	0.029	−0.002	0.005	0.001	PASS
Flourishing	Strict	288.17	269	0.998	0.997	0.014	[0.000, 0.026]	0.029	0.0	0.002	0.0	PASS

Chen (2007) criteria: Metric vs Configural: Delta-CFI > = −0.010, Delta-RMSEA <= 0.015, Delta-SRMR <= 0.030; Scalar vs Metric: Delta-CFI > = −0.010, Delta-RMSEA <= 0.015, Delta-SRMR <= 0.010. All scales achieved scalar invariance.

### 3.5 Longitudinal mediation model: primary analysis

The core longitudinal mediation SEM (Figure 2) showed mixed or borderline fit: Satorra–Bentler scaled χ^2^(15) = 56.38, *p* < 0.001, robust CFI = 0.962, robust TLI = 0.910, robust RMSEA = 0.090, 90% CI [0.065, 0.117], and SRMR = 0.063. The CFI met the commonly used criterion of 0.90, whereas the RMSEA exceeded the conventional threshold of 0.08 and the TLI fell below the preferred criterion of 0.95. The overall model fit was therefore considered mixed rather than uniformly good. These indices were obtained using robust maximum likelihood estimation with Satorra–Bentler scaling. By contrast, the competing-model comparisons reported in Table 6 used standard ML χ^2^ values for nested-model testing, as required for Satorra–Bentler difference testing in lavaan.

**Figure 2:**
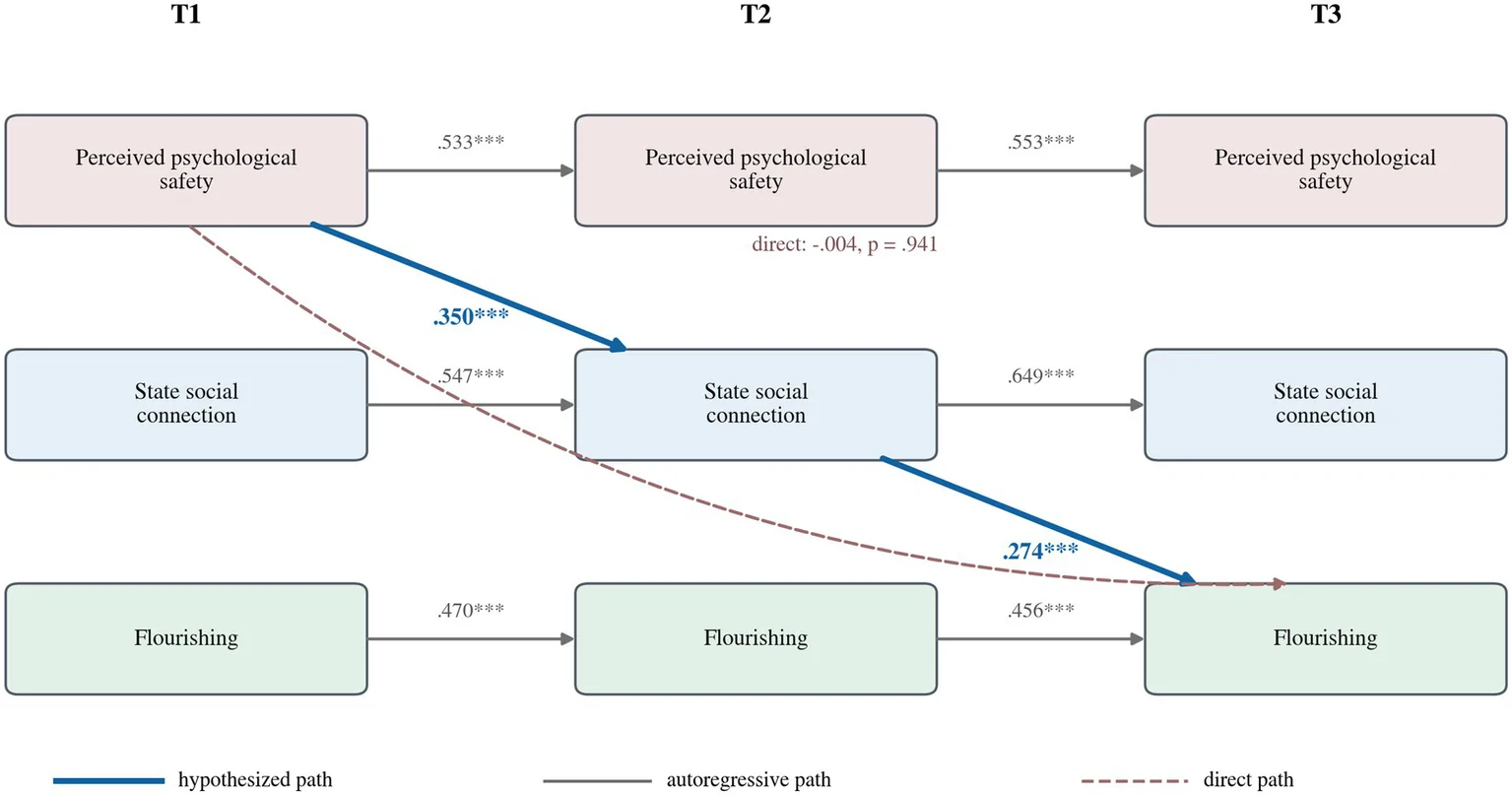
Primary longitudinal mediation model with standardized coefficients (*N* = 341). Dark blue arrows show the hypothesized indirect pathway, gray arrows show autoregressive paths, and the dashed arrow shows the direct path. Baseline adjustment paths and within-wave covariances were estimated but omitted from the figure for readability; all estimates are reported in Table 7. *** *p* < 0.001.

**Table 6:** Competing model comparison.

Model	Chi^2^	df	*p*	CFI	TLI	RMSEA	RMSEA_CI	SRMR	AIC	BIC	Heywood	Note	Delta_AIC	Delta_BIC
Hypothesized (PS- > SC- > FL)	52.19	15	< 0.001	0.967	0.921	0.085	[0.061, 0.111]	0.063	8251.9	8401.4	False		0.0	0.0
Reverse (PS- > FL- > SC)	166.84	15	< 0.001	0.866	0.678	0.172	[0.149, 0.196]	0.107	8366.6	8516.0	False		114.7	114.6
Alternative (SC- > PS- > FL)	128.96	15	< 0.001	0.899	0.759	0.149	[0.126, 0.174]	0.081	8328.7	8478.2	False		76.8	76.8
Reciprocal (reduced cross-lagged)	44.61	13	< 0.001	0.972	0.923	0.084	[0.058, 0.112]	0.052	8248.4	8405.5	False		−3.5	4.1

All four models used the same nine observed variables, autoregressive backbone, wave-specific covariance structure, baseline adjustment logic, ML/FIML estimator, and analytic sample. AIC favored the reciprocal model, whereas BIC favored the more parsimonious hypothesized model; the evidence is therefore mixed.

The path coefficients presented in Table 7 supported the main hypotheses. H1 was supported: T1 perceived psychological safety was positively associated with T2 state social connection, B = 0.3608, SE = 0.0387, *z* = 9.32, *p* < 0.001, *β* = 0.350. Students who reported greater perceived psychological safety in collaborative music activities also reported stronger state social connection 6 weeks later. H2 was also supported: T2 state social connection was positively associated with T3 flourishing, B = 0.2629, SE = 0.0536, *z* = 4.90, *p* < 0.001, *β* = 0.274. Students who reported a stronger sense of connection subsequently showed higher levels of flourishing (Table 8).

**Table 7:** Longitudinal mediation model: standardized and unstandardized path coefficients.

Path	Label	Est_Unstd	SE	*z*	*p*	CI_Lower	CI_Upper	Est_Std
T2_PS_mean <− T1_PS_mean	ps_ar	0.5388	0.0457	11.8	< 0.001	0.4493	0.6282	0.5331
T3_PS_mean <− T2_PS_mean		0.5517	0.0432	12.771	< 0.001	0.467	0.6363	0.5528
T2_SC_mean <− T1_SC_mean	sc_ar	0.5666	0.0387	14.649	< 0.001	0.4908	0.6424	0.5466
T3_SC_mean <− T2_SC_mean		0.6371	0.0412	15.453	< 0.001	0.5563	0.7179	0.649
T2_FL_mean <− T1_FL_mean	fl_ar	0.4641	0.0624	7.438	< 0.001	0.3418	0.5864	0.47
T3_FL_mean <− T2_FL_mean		0.4421	0.0427	10.359	< 0.001	0.3585	0.5258	0.4556
T2_SC_mean <− T1_PS_mean	a_path	0.3608	0.0387	9.316	< 0.001	0.2849	0.4368	0.3504
T3_FL_mean <− T2_SC_mean	b_path	0.2629	0.0536	4.901	< 0.001	0.1578	0.368	0.2737
T3_FL_mean <− T1_PS_mean	cprime_path	−0.0036	0.0488	−0.074	0.9413	−0.0993	0.0921	−0.0036
T2_SC_mean <− T1_FL_mean		−0.0571	0.0438	−1.304	0.1924	−0.143	0.0288	−0.0572
T3_FL_mean <− T1_FL_mean		0.1969	0.0448	4.399	< 0.001	0.1092	0.2846	0.2055
T3_FL_mean <− T1_SC_mean		0.0045	0.0552	0.082	0.9345	−0.1036	0.1127	0.0046
indirect	indirect	0.0949	0.0219	4.322	< 0.001	0.0518	0.1379	0.0959
total	total	0.0913	0.0456	2.0	0.046	0.0018	0.1807	0.0923

MLR estimator with FIML. Model fit: scaled chi^2^(15) = 56.38, *p* < 0.001, robust CFI = 0.962, robust TLI = 0.910, robust RMSEA = 0.090 [[0.065, 0.117]], SRMR = 0.063. Beta, standardized coefficient.

**Table 8:** Summary of direct, indirect, and total effects (mediation model).

Effect	Hypothesis	Est	SE	*p*	CI_Lower	CI_Upper
a_path (T1 PS - > T2 SC)	H1	0.3608	0.0387	< 0.001	0.2849	0.4368
b_path (T2 SC - > T3 FL)	H2	0.2629	0.0536	< 0.001	0.1578	0.368
c’ (T1 PS - > T3 FL, direct)	Direct effect	−0.0036	0.0488	0.9413	−0.0993	0.0921
Indirect (a*b)	H3	0.0949	0.0219	< 0.001	0.0518	0.1379
Total effect	Total	0.0913	0.0456	0.046	0.0018	0.1807

Delta-method standard errors. The total-effect p value is reported as 0.046 after rounding to three decimals.

H3, the central mediation hypothesis, was supported. The indirect effect was significant, B = 0.0949, SE = 0.0219, *p* < 0.001, *β* = 0.096. The direct effect was not significant, B = −0.0036, SE = 0.0488, *p* = 0.941, *β* = −0.004. The total effect was small and close to the conventional threshold, B = 0.0913, SE = 0.0456, *p* = 0.046, 95% CI [0.002, 0.181], *β* = 0.092. The indirect association was therefore more stable than either the direct or total association.

### 3.6 Bootstrap test of the indirect effect

To address the limitations of the delta method in finite samples, the indirect effect was further examined using 5,000 bootstrap samples with bias-corrected and accelerated confidence intervals (Table 9). Because bootstrap estimation is not supported with the MLR estimator, the model was re-estimated using maximum likelihood estimation. The indirect effect remained significant, B = 0.0949, SE = 0.0224, 95% BCa CI [0.054, 0.142]. Because the confidence interval did not include zero, H3 received further support. The confidence interval for the direct effect included zero, consistent with the primary analysis.

**Table 9:** Bootstrap indirect effects (5,000 Draws, BCa confidence intervals).

Parameter	Est	SE	*z*	*p*	CI_Lower	CI_Upper	Sig
a_path (T1 PS - > T2 SC) [H1]	0.3608	0.0388	9.309	< 0.001	0.2838	0.4366	YES (CI excludes 0)
b_path (T2 SC - > T3 FL) [H2]	0.2629	0.0542	4.854	< 0.001	0.1517	0.3663	YES (CI excludes 0)
indirect (a*b) [H3]	0.0949	0.0224	4.232	< 0.001	0.0545	0.142	YES (CI excludes 0)

ML estimator. BCa CI = Bias-corrected and accelerated confidence interval. CI excluding 0 is considered significant at alpha = 0.05.

### 3.7 Comparison of competing models

Table 6 compares four models estimated under directly comparable specifications. The hypothesized model fit substantially better than the reverse model (Delta AIC = 114.7) and the alternative-ordering model (Delta AIC = 76.8). These comparisons indicate that those two alternative sequences were poorly supported relative to the hypothesized model. They do not, however, establish that the hypothesized ordering is uniquely or causally correct.

The reciprocal model had an AIC 3.5 points lower than the hypothesized model, whereas its BIC was 4.1 points higher. The information criteria therefore gave mixed evidence: AIC favored the reciprocal specification and BIC favored parsimony. Moreover, the added path from earlier state social connection to later perceived psychological safety was significant (*β* = 0.134, *p* = 0.004), while the added flourishing-to-connection path was not (*p* = 0.419). The focal perceived-safety-to-connection path remained significant. Taken together, the results support the hypothesized indirect pathway but do not justify presenting perceived psychological safety as clearly prior to social connection. A reciprocal reinforcing process is also compatible with the data.

### 3.8 RI-CLPM robustness analysis

To further distinguish between-person and within-person effects, a random-intercept cross-lagged panel model (RI-CLPM; Hamaker et al., 2015) was estimated as a robustness analysis. The RI-CLPM separates the observed variance into stable between-person components, represented by random intercepts, and time-varying within-person components.

The RI-CLPM showed good fit, χ^2^(15) = 23.86, *p* = 0.068, CFI = 0.992, TLI = 0.981, RMSEA = 0.042, 90% CI [0.000, 0.072], and SRMR = 0.033. All random-intercept variances were greater than zero, and no Heywood cases or negative variances were observed. The parameter estimates were within acceptable ranges. However, the three-wave RI-CLPM had relatively few degrees of freedom (df = 15) and was close to saturation, which may have inflated the CFI and TLI values. A three-wave design also represents the minimum number of waves required for RI-CLPM estimation, and the standard errors of the within-person parameters may therefore be relatively large (Orth et al., 2021). The RI-CLPM results should thus be interpreted as exploratory robustness evidence rather than as definitive evidence of a within-person causal mechanism.

At the within-person level, after the autoregressive paths were constrained to equality across intervals, the indirect effect was significant, B = 0.1697, SE = 0.0490, *p* < 0.001, *β* = 0.177. The total effect was not significant, B = 0.0550, *p* = 0.446.

The within-person pattern obtained from the RI-CLPM differed from that of the standard SEM. In the standard SEM, the total effect was small and statistically significant, B = 0.0913, *p* = 0.046, whereas the within-person total effect in the RI-CLPM was not significant, B = 0.0550, *p* = 0.446. Only the within-person indirect effect remained significant, B = 0.1697, *p* < 0.001. This contrast suggests that the total effect in the standard model may have been partly influenced by stable between-person differences. After these differences were separated, the perceived psychological safety → state social connection → flourishing pathway showed a significant indirect effect but no significant total effect at the within-person level. These findings should nevertheless be interpreted cautiously because a three-wave design provides limited parameter identification for separating within-person and between-person processes (Orth et al., 2021; Usami et al., 2019) (Tables 10, 11).

**Table 10:** RI-CLPM within-person path coefficients (robustness check).

Parameter	Est	SE	*p*	Beta	Admissible	Interpretation
a_wp (W_SC2 < - W_PS1) [H1 within-person]	0.4341	0.0671	< 0.001	0.4186	True	Admissible
b_wp (W_FL3 < - W_SC2) [H2 within-person]	0.391	0.0788	< 0.001	0.4223	True	Admissible
c_wp (W_FL3 < - W_PS1) [direct within-person]	−0.1147	0.0856	0.1803	−0.1195	True	Admissible
wp_indirect (a_wp * b_wp) [H3 within-person]	0.1697	0.049	< 0.001	0.1768	True	Admissible
wp_total (c_wp + indirect)	0.055	0.0722	0.4462	0.0573	True	Admissible
wp_ar_ps (PS autoregression)	0.253	0.094	0.0071	0.2536	True	Admissible
wp_ar_sc (SC autoregression)	0.3902	0.0935	< 0.001	0.3624	True	Admissible
wp_ar_fl (FL autoregression)	0.2701	0.0999	0.0069	0.2671	True	Admissible

RI-CLPM decomposes variance into between-person (random intercepts) and within-person components. Autoregressive paths constrained equal across lag-pairs for model parsimony. The three-wave design limits parameter identifiability; close-to-saturated model may inflate CFI/TLI.

**Table 11:** CLPM and RI-CLPM model fit comparison.

Model	Chi^2^	df	*p*	CFI	TLI	RMSEA	RMSEA_CI	SRMR	AIC	BIC	Note
CLPM	77.59	21	0.0	0.95	0.914	0.089	[0.068, 0.110]	0.066	8265.3	8391.8	
RI-CLPM	23.86	15	0.0675	0.992	0.981	0.042	[0.000, 0.072]	0.033	8223.6	8373.1	

CLPM, Cross-Lagged Panel Model; RI-CLPM, Random Intercept CLPM. Standard CLPM fit: chi^2^(21) = 77.6, CFI = 0.950, RMSEA = 0.089.

### 3.9 Control variables and auxiliary analyses

Regression analyses showed that age, gender, and years of music training did not significantly predict or correlate with the core constructs and were not included in the primary model. T1 collaborative music activity characteristics (CM) correlated with T1 perceived psychological safety (*r* = 0.451) and T1 state social connection (*r* = 0.402). CM was conceptualized as a potential baseline contextual antecedent or covariate rather than as a mediator or moderator.

A sensitivity model added T1 CM as a baseline covariate. The indirect estimate remained similar (B = 0.092, *p* < 0.001) to the primary estimate (B = 0.0949, *p* < 0.001), although overall fit was poorer (CFI = 0.899, RMSEA = 0.138, SRMR = 0.125). The poorer fit is reported as a diagnostic result, not as the reason for excluding CM from the confirmatory model. The confirmatory estimand concerned the association between perceived safety and later connection; adjusting for a concurrently measured, self-developed measure with uncertain causal ordering could produce overadjustment or construct overlap. CM also showed low temporal correlations (T1-T2 *r* = 0.122; T2-T3 *r* = 0.207), which may reflect genuinely time-varying collaborative behavior, measurement instability, or both. These possibilities cannot be distinguished with the present measure and are treated as a limitation.

### 3.10 Timing and missing-data sensitivity analyses

Survey completion times were recorded for all participants. The T1-T2 interval was M = 42.0 days (SD = 7.0), and the T2-T3 interval was M = 42.2 days (SD = 7.1); both ranged from 28 to 56 days. The six-week window was intended to span repeated rehearsal and feedback cycles while retaining a reasonably comparable activity context. It was not selected because prior evidence established a six-week causal lag. Timing covariates were therefore examined as a sensitivity analysis.

In the first analysis, the number of days between T1 and T2 and between T2 and T3 was included as covariates in the primary model. Model fit improved (CFI = 0.973, RMSEA = 0.054), and the indirect effect remained significant, B = 0.0917, *p* < 0.001. The total effect was also significant, B = 0.0902, *p* = 0.050. These results indicate that the mediation pathway remained robust after variation in assessment intervals was controlled.

In the second analysis, restricting the sample to participants whose survey intervals fell between 28 and 56 days produced the same sample as the primary analysis because all 341 participants met this criterion. The results were therefore identical to those of the primary model and are not reported separately. Together, the two analyses indicate that variation in survey intervals did not threaten the main mediation findings.

Wave-specific availability was not associated with prior observed scores. Participants with versus without T2 group-context measures did not differ in T1 perceived psychological safety (*p* = 0.927), state social connection (*p* = 0.264), or flourishing (*p* = 0.725). Participants with versus without T3 group-context measures did not differ in T2 perceived psychological safety (*p* = 0.844), state social connection (*p* = 0.910), or flourishing (*p* = 0.279). In a regression-based complete-case analysis (*N* = 306), the a path was B = 0.349, the b path was B = 0.268, and the indirect effect was B = 0.094, bootstrap 95% CI [0.052, 0.143]. In an inverse-probability-weighted analysis for T2 availability, the corresponding estimates were B = 0.372, B = 0.268, and B = 0.100. These estimates were close to the primary FIML results, but selection related to unobserved safety or connection cannot be excluded.

Because the data had a nested structure, with students clustered within 23 music groups, the primary model was also re-estimated using cluster-robust standard errors based on GROUP_ID. The ratios of cluster-robust standard errors to MLR standard errors were close to 1.0, ranging from 0.81 to 1.06, as shown in Table 12. The significance of the key parameters remained unchanged. The indirect effect remained significant, B = 0.0949, cluster-robust SE = 0.0225, *p* < 0.001, and the total effect remained significant after cluster adjustment, B = 0.0913, cluster-robust SE = 0.0372, *p* = 0.014. The standard-error ratios close to 1.0 indicate minimal within-group dependence and suggest that clustering at the music-group level had little influence on the estimated uncertainty.

**Table 12:** Cluster-robust standard error comparison (GROUP_ID, 23 clusters).

Parameter	Est	SE_MLR	p_MLR	SE_Cluster	p_Cluster	SE_Ratio
a_path (T1 PS → T2 SC)	0.3608	0.0387	< 0.001	0.0362	< 0.001	0.934
b_path (T2 SC → T3 FL)	0.2629	0.0536	< 0.001	0.0571	< 0.001	1.064
c’ (Direct effect)	−0.0036	0.0488	0.941	0.0457	0.937	0.936
Indirect (a × b)	0.0949	0.0219	< 0.001	0.0225	< 0.001	1.026
Total (c′ + a × b)	0.0913	0.0456	0.046	0.0372	0.014	0.815

SE_MLR = standard MLR robust SE (no clustering). SE_Cluster = cluster-robust SE with GROUP_ID. SE_Ratio = SE_Cluster / SE_MLR. Ratio near 1.0 indicates negligible within-cluster dependency.

## 4 Discussion

### 4.1 Main findings

This study used a three-wave longitudinal design to examine whether individually perceived psychological safety was associated with later flourishing through state social connection in collaborative music activities. The hypothesized indirect pathway was significant across the primary, bootstrap, complete-case, weighted, cluster-robust, and exploratory RI-CLPM analyses. The direct effect was near zero. However, the reciprocal model had a lower AIC, and earlier connection significantly predicted later perceived safety. The evidence therefore supports an indirect association while leaving a reciprocal safety-connection process plausible.

The results show that participation in a choir, ensemble, or group class should not be treated as uniformly beneficial. Students who personally perceived that they could acknowledge mistakes, ask questions, seek help, and express different artistic views later reported stronger connection, which was associated with later flourishing. This is evidence for a general interpersonal process observed in music education. Because synchrony, collective expression, and shared emotion were not measured, the findings do not establish that a uniquely musical mechanism generated the pathway.

### 4.2 The role of psychological safety in collaborative music activities

The association between earlier perceived psychological safety and later social connection extends the individual-level application of psychological safety to higher music education. Research on project-based learning and interdisciplinary student teams has shown that when students believe they can express disagreement, reveal uncertainty, or try new approaches without negative social consequences, they are more willing to share knowledge, engage in problem-solving, and take creative risks (Han et al., 2022; Moffett et al., 2024; Robinson and Held, 2025).

Kahn's (1990) engagement framework and Edmondson's (1999) team-learning account help clarify why perceived safety may matter in this setting. Both emphasize that safety reduces the interpersonal cost of self-expression. Frazier et al.'s (2017) meta-analysis further positions psychological safety within a broader network of supportive relationships, information sharing, learning behavior, and performance. Applied to collaborative music, this perspective suggests that safety is not simply a pleasant emotional climate; it is a condition that may allow students to expose incomplete interpretations, acknowledge errors, and remain actively engaged in joint artistic problem-solving.

This general process is salient in collaborative music activities because mistakes can be publicly audible and students must accept correction while adjusting their performance to others. Fear of negative evaluation may encourage silence, concealment, or passive compliance. Conversely, a student’s perception that interpersonal risk is tolerable may make honest feedback and reciprocal response more likely. The study measured these individual perceptions and later relational experience, but it did not measure the musical interaction mechanisms through which they may have developed.

The findings also highlight the need to distinguish demanding artistic standards from a student’s perception of interpersonal threat. The present data do not show that any music group possessed a shared safe climate: perceived psychological safety was measured and analyzed at the student level, and ICC values were low. The results therefore concern differences in students’ appraisals within and across collaborative settings. Future multilevel studies should measure and justify any aggregation of shared group beliefs.

### 4.3 The mediating role of state social connection

State social connection significantly predicted later flourishing, indicating that immediate relational experiences within collaborative music activities may have psychological significance beyond a single rehearsal. State social connection captures feelings of closeness, acceptance, and belonging within a particular period and context. It is therefore more sensitive to changes in a specific group environment than general social support or relatively stable interpersonal tendencies (Lok and Dunn, 2023).

The proposed mediating role of state social connection can also be situated within self-determination theory. Ryan and Deci (2000) identify relatedness as a basic psychological need whose satisfaction supports motivation and well-being. A psychologically safe interaction may make relatedness more attainable by allowing students to participate without self-protective withdrawal, while state social connection captures the immediate experience that this need has been met. Within positive psychology, Keyes (2002) and Diener et al. (2010) conceptualize flourishing as positive functioning that includes meaning, competence, engagement, optimism, and supportive relationships. The model therefore links a local interpersonal appraisal to a proximal experience of relatedness and, subsequently, to broader positive functioning.

When students receive responses, recognition, and confirmation of their membership within a group, they may be more likely to feel that their participation matters. Completing a musical work together may also strengthen competence, meaning, and positive relationships. These experiences correspond closely to the purpose, relationships, engagement, and positive self-evaluation represented in flourishing. Previous research has reported stable associations between social connection, prosocial orientation, and flourishing. Social development, value development, and learning support have also been linked to eudaimonic and social well-being among university students (Rahe and Jansen, 2023; Zhao and Hua, 2025).

The indirect-effect pattern is consistent with perceived psychological safety as one possible antecedent of later social connection, but it does not establish a fixed one-way mechanism. A student’s sense that interpersonal risk is tolerable may facilitate genuine communication and reciprocal response. At the same time, positive social experiences may strengthen later perceptions of safety, as indicated by the significant reverse path in the reciprocal model.

The total effect was small and close to the conventional significance threshold, so it is not treated as the central result. The more consistent finding was the indirect association across sensitivity analyses. Its theoretical contribution is a contextually bounded test of a general group-process account in higher music education, not evidence that collaborative music has a unique causal effect on flourishing.

### 4.4 Temporal ordering and possible reciprocal relationships

The reverse and alternative-ordering models fit substantially worse than the hypothesized model, but the reciprocal model yielded a lower AIC and a slightly higher BIC. Consequently, the longitudinal evidence does not clearly privilege a strictly unidirectional sequence. Model comparisons show relative compatibility with the observed covariance structure; they do not establish causal priority.

In the reciprocal model, earlier state social connection significantly predicted later perceived psychological safety. A plausible interpretation is reinforcement: feeling able to take interpersonal risks may support closeness and belonging, while positive social experiences may subsequently reduce concerns about negative evaluation. More waves, stronger time-varying measures, and experimental or quasi-experimental designs are needed to test this dynamic directly.

The RI-CLPM also identified a significant indirect effect at the within-person level. This suggests that when a student’s psychological safety was higher than their usual level, later social connection and flourishing may also have changed accordingly. However, the study included only three waves, placing the RI-CLPM close to the minimum requirements for identification. These findings should therefore be treated as exploratory. They do not replace experimental evidence and are not sufficient to show that increasing psychological safety necessarily causes improvements in flourishing.

### 4.5 Practical implications

The findings suggest that music teachers and conductors should attend to students’ perceptions of rehearsal interaction as well as task completion and performance quality. Because the evidence is observational and individual-level, the following practices are implications to be tested rather than proven interventions. Teachers can model openness about uncertainty, address mistakes through specific guidance rather than humiliation, invite students to explain difficulties, and respond seriously to different artistic interpretations.

Peer feedback should maintain both professional standards and relational support. Teachers may use structured feedback procedures in which students first describe what they heard and then offer a practical suggestion, reducing the risk that technical comments are interpreted as judgments of personal ability. Small-group discussion, rotating leadership, collaborative composition, and short post-rehearsal reflections may also create more opportunities for students to express views and respond to one another. Research on student teams in higher education has shown that clear interactional norms, instructor modeling, and organized communication practices can support psychological safety and knowledge sharing (Moffett et al., 2024; Robinson and Held, 2025).

The practical goal should extend beyond reducing tension or avoiding conflict. Perceived safety needs to be translated into meaningful relational experiences. Teachers may consider whether peripheral members are given opportunities to speak, whether students can collaborate beyond established friendship groups, and whether feedback leads to genuine mutual understanding. Recent research in music education has similarly identified teacher support, peer relationships, and social connection as important elements through which music participation may contribute to well-being and resilience (de Bruin, 2025).

## 5 Conclusion

Across three waves, individually perceived psychological safety was associated with later flourishing through stronger state social connection among university students in collaborative music activities. The indirect pathway was robust, but competing-model evidence also supported a plausible reciprocal relation between perceived safety and connection. The study therefore provides a contextually bounded examination of a general interpersonal process in higher music education. It does not establish causal priority or a uniquely musical mechanism.

Several limitations qualify the findings. The observational design does not establish causality, and the primary model showed mixed fit. AIC favored the reciprocal model while BIC favored the more parsimonious model. Psychological safety was measured as an individual perception, so no claim can be made about a shared team-level construct. Although cluster-robust standard errors addressed dependence, only 23 clusters were available and no multilevel group-level mechanism was tested. All core variables were self-reported.

The study also pooled heterogeneous activity types whose leadership structures, group sizes, hierarchies, and interaction demands may differ. Synchrony, collective expression, and shared emotion were discussed as contextual features but were not measured. The self-developed CM measure had high internal consistency but low temporal stability and uncertain causal ordering. Nonmonotonic missingness was explained by wave-specific participation status, and observed-data sensitivity analyses were reassuring, but FIML cannot eliminate bias if missingness depended on unobserved safety or connection. Finally, the unusual lagged correlations were verified against the item-level data but warrant independent replication.

Future research should use validated measures of music-specific interaction, compare activity types, and collect enough groups for multilevel analysis of shared psychological safety. Four or more waves would better separate within-person and between-person processes and model reciprocity. Experimental or quasi-experimental studies could test whether changes in teaching and rehearsal practices alter perceived safety, connection, and flourishing. Future studies should also retain detailed transition reasons and use observational, peer, teacher, and behavioral indicators alongside self-report.

## Data Availability

The original contributions presented in the study are included in the article/Supplementary material, further inquiries can be directed to the corresponding author.
