# Supplementary material for: Perceived psychological safety, state social connection, and student flourishing in collaborative music activities: a three-wave longitudinal study
Source: Front Psychol. 2026 Sep 25;17:1956682. doi: 10.3389/fpsyg.2026.1956682 (PMC13649971; doi:10.3389/fpsyg.2026.1956682)
Supplement: Supplementary file 1 [file Supplementary_file_1.docx]

Supplementary Material

**Perceived Psychological Safety, State Social Connection, and Student Flourishing in Collaborative Music Activities**

SHIHAO ZHANG

# Supplementary Table S1. Spearman Correlation Matrix

| **Variable** | **1** | **2** | **3** | **4** | **5** | **6** | **7** | **8** | **9** | **10** | **11** | **12** | **13** | **14** | **15** |
| --- | --- | --- | --- | --- | --- | --- | --- | --- | --- | --- | --- | --- | --- | --- | --- |
| 1. PS T1 | 1.000 |  |  |  |  |  |  |  |  |  |  |  |  |  |  |
| 2. PS T2 | .539*** | 1.000 |  |  |  |  |  |  |  |  |  |  |  |  |  |
| 3. PS T3 | .395*** | .545*** | 1.000 |  |  |  |  |  |  |  |  |  |  |  |  |
| 4. SC T1 | .426*** | .342*** | .261*** | 1.000 |  |  |  |  |  |  |  |  |  |  |  |
| 5. SC T2 | .577*** | .362*** | .252*** | .649*** | 1.000 |  |  |  |  |  |  |  |  |  |  |
| 6. SC T3 | .348*** | .338*** | .200* | .501*** | .647*** | 1.000 |  |  |  |  |  |  |  |  |  |
| 7. FL T1 | .288*** | .185* | .174 | .372*** | .235** | .135 | 1.000 |  |  |  |  |  |  |  |  |
| 8. FL T2 | .293*** | .225** | .163 | .258*** | .235** | .136 | .500*** | 1.000 |  |  |  |  |  |  |  |
| 9. FL T3 | .351*** | .292*** | .206* | .361*** | .420*** | .177 | .457*** | .581*** | 1.000 |  |  |  |  |  |  |
| 10. CM T1 | .451*** | .246*** | .177 | .402*** | .378*** | .238*** | .234*** | .148 | .205** | 1.000 |  |  |  |  |  |
| 11. CM T2 | .292*** | .368*** | .242** | .258*** | .365*** | .308*** | .050 | .031 | .142 | .122 | 1.000 |  |  |  |  |
| 12. CM T3 | .265*** | .286*** | .362*** | .260*** | .305*** | .310*** | .125 | .129 | .080 | .149 | .207* | 1.000 |  |  |  |
| 13. O T3 | .197* | .282*** | .208** | .311*** | .353*** | .415*** | .254*** | .188* | .406*** | .199* | .158 | .109 | 1.000 |  |  |
| 14. Age | -.020 | .028 | .034 | -.019 | -.019 | -.062 | -.072 | -.079 | -.085 | -.025 | -.054 | -.009 | -.071 | 1.000 |  |
| 15. Music years | .028 | .039 | -.033 | .012 | -.034 | -.044 | .080 | .042 | .041 | .056 | -.021 | .019 | -.017 | .019 | 1.000 |

Note: Lower triangle shown. Pairwise-complete Spearman correlations. Holm-Bonferroni adjusted significance: * p < .05, ** p < .01, *** p < .001. PS = perceived psychological safety; SC = state social connection; FL = flourishing; CM = collaborative music activity characteristics; O = continued participation intention.

# Supplementary Table S2. Pairwise Sample Sizes for the Correlation Matrix

| **Variable** | **1** | **2** | **3** | **4** | **5** | **6** | **7** | **8** | **9** | **10** | **11** | **12** | **13** | **14** | **15** |
| --- | --- | --- | --- | --- | --- | --- | --- | --- | --- | --- | --- | --- | --- | --- | --- |
| 1. PS T1 | 341 |  |  |  |  |  |  |  |  |  |  |  |  |  |  |
| 2. PS T2 | 324 | 324 |  |  |  |  |  |  |  |  |  |  |  |  |  |
| 3. PS T3 | 322 | 306 | 322 |  |  |  |  |  |  |  |  |  |  |  |  |
| 4. SC T1 | 341 | 324 | 322 | 341 |  |  |  |  |  |  |  |  |  |  |  |
| 5. SC T2 | 324 | 324 | 306 | 324 | 324 |  |  |  |  |  |  |  |  |  |  |
| 6. SC T3 | 322 | 306 | 322 | 322 | 306 | 322 |  |  |  |  |  |  |  |  |  |
| 7. FL T1 | 341 | 324 | 322 | 341 | 324 | 322 | 341 |  |  |  |  |  |  |  |  |
| 8. FL T2 | 341 | 324 | 322 | 341 | 324 | 322 | 341 | 341 |  |  |  |  |  |  |  |
| 9. FL T3 | 341 | 324 | 322 | 341 | 324 | 322 | 341 | 341 | 341 |  |  |  |  |  |  |
| 10. CM T1 | 341 | 324 | 322 | 341 | 324 | 322 | 341 | 341 | 341 | 341 |  |  |  |  |  |
| 11. CM T2 | 324 | 324 | 306 | 324 | 324 | 306 | 324 | 324 | 324 | 324 | 324 |  |  |  |  |
| 12. CM T3 | 322 | 306 | 322 | 322 | 306 | 322 | 322 | 322 | 322 | 322 | 306 | 322 |  |  |  |
| 13. O T3 | 341 | 324 | 322 | 341 | 324 | 322 | 341 | 341 | 341 | 341 | 324 | 322 | 341 |  |  |
| 14. Age | 341 | 324 | 322 | 341 | 324 | 322 | 341 | 341 | 341 | 341 | 324 | 322 | 341 | 341 |  |
| 15. Music years | 341 | 324 | 322 | 341 | 324 | 322 | 341 | 341 | 341 | 341 | 324 | 322 | 341 | 341 | 341 |

Note: Pairwise N varies because group-context measures were skipped according to wave-specific participation status. Flourishing, age, and music-training years were available for all 341 participants.

# Supplementary Table S3. Convergent Validity and Composite Reliability

| **Wave** | **Factor** | **AVE** | **Square root of AVE** | **CR** |
| --- | --- | --- | --- | --- |
| T1 | PS | 0.673 | 0.821 | 0.935 |
| T1 | SC | 0.694 | 0.833 | 0.958 |
| T1 | FL | 0.741 | 0.861 | 0.958 |
| T2 | PS | 0.717 | 0.847 | 0.947 |
| T2 | SC | 0.704 | 0.839 | 0.959 |
| T2 | FL | 0.722 | 0.85 | 0.954 |
| T3 | PS | 0.721 | 0.849 | 0.947 |
| T3 | SC | 0.701 | 0.837 | 0.959 |
| T3 | FL | 0.712 | 0.844 | 0.952 |

Note: AVE = average variance extracted; CR = composite reliability. All AVE values exceed .50 and all CR values exceed .70.

# Supplementary Table S4. Missingness Diagnostics and Sensitivity Analyses

Panel A. Wave-specific participation statuses that triggered omission of the group-context block

| **Reason** | **T2 n** | **T3 n** |
| --- | --- | --- |
| Temporarily stopped | 7 | 6 |
| Fully withdrew | 4 | 12 |
| Changed group | 6 | 1 |
| Total skipped | 17 | 19 |

Panel B. Prior scores by subsequent availability of group-context measures

| **Availability contrast** | **Prior score** | **Observed-block M** | **Skipped-block M** | **p** | **Cohen d** |
| --- | --- | --- | --- | --- | --- |
| T2 available vs skipped | T1 perceived psychological safety | 3.983 | 4.008 | .927 | -.022 |
| T2 available vs skipped | T1 state social connection | 3.984 | 4.259 | .264 | -.246 |
| T2 available vs skipped | T1 flourishing | 3.940 | 4.051 | .725 | -.096 |
| T3 available vs skipped | T2 perceived psychological safety | 4.002 | 4.048 | .844 | -.040 |
| T3 available vs skipped | T2 state social connection | 4.020 | 3.989 | .910 | .027 |
| T3 available vs skipped | T2 flourishing | 3.965 | 4.289 | .279 | -.282 |

Panel C. Mediation sensitivity estimates

| **Analysis** | **N** | **a path B** | **b path B** | **Indirect B** | **95% CI** |
| --- | --- | --- | --- | --- | --- |
| Primary FIML SEM | 341 | .3608 | .2629 | .0949 | [.054, .142] |
| Complete-case regressions | 306 | .3491 | .2681 | .0936 | [.052, .143] |
| Inverse-probability weighted regressions | 324 | .3723 | .2677 | .0997 | Not estimated |

Note: The complete-case and weighted results are regression-based diagnostics rather than exact substitutes for the primary FIML SEM. The complete-case confidence interval is percentile-bootstrap based on 5,000 resamples. These analyses address selection on observed variables but cannot rule out missing-not-at-random selection on unobserved experiences.

# Supplementary Note S5. Exploratory Analysis of Continued Participation Intention

Continued participation intention was measured only at T3 using four seven-point items (M = 4.008, SD = 1.119; Cronbach's alpha = .876). Because it was not part of the preregistered longitudinal sequence and was measured at the same wave as its main correlates, this analysis is exploratory and primarily cross-sectional.

T3 continued participation intention was positively correlated with T3 state social connection and T3 flourishing. In the simultaneous regression reported in the original analysis, T3 flourishing (β = .360, p < .001) and T3 state social connection (β = .311, p < .001) were positive predictors of continued participation intention (R² = .287). T1 perceived psychological safety was not significant in that model (β = -.120, p = .078). These results are not used to support H1-H3 and should not be interpreted causally.
